# Interactions between Vitamin D Genetic Risk and Dietary Factors on Metabolic Disease-Related Outcomes in Ghanaian Adults

**DOI:** 10.3390/nu14132763

**Published:** 2022-07-04

**Authors:** Buthaina E. Alathari, David A. Nyakotey, Abdul-Malik Bawah, Julie A. Lovegrove, Reginald A. Annan, Basma Ellahi, Karani S. Vimaleswaran

**Affiliations:** 1 Hugh Sinclair Unit of Human Nutrition, Department of Food and Nutritional Sciences, Harry Nursten Building, Pepper Lane, University of Reading, Reading RG6 6DZ, UK; b.e.a.a.alathari@pgr.reading.ac.uk (B.E.A.); j.a.lovegrove@reading.ac.uk (J.A.L.); 2 Department of Food Science and Nutrition, Faculty of Health Sciences, The Public Authority for Applied Education and Training, P.O. Box 14281, AlFaiha 72853, Kuwait; 3 Department of Biochemistry and Biotechnology, College of Science, Kwame Nkrumah University of Science and Technology, Accra Road, Kumasi GH233, Ghana; dnyakotey@gmail.com (D.A.N.); malikbawa2008@yahoo.com (A.-M.B.); reggie@imtf.org (R.A.A.); 4 Liggins Institute, University of Auckland, 85 Park Road, Grafton, Auckland 1023, New Zealand; 5 Institute of Cardiovascular and Metabolic Research, Harry Nursten Building, Pepper Lane, University of Reading, Reading RG6 6DZ, UK; 6 Faculty of Health and Social Care, University of Chester, Riverside Campus, Chester CH1 4BJ, UK; b.ellahi@chester.ac.uk; 7 Institute for Food, Nutrition and Health, University of Reading, Reading RG6 6AH, UK

**Keywords:** Ghana, gene–diet interaction, genetic risk score, metabolic traits, fiber, fat

## Abstract

The Ghanaian population is experiencing an upsurge in obesity and type 2 diabetes (T2D) due to rapid urbanization. Besides dietary factors, vitamin D-related genetic determinants have also been shown to contribute to the development of obesity and T2D. Hence, we aimed to examine the interactions between dietary factors and vitamin D-related genetic variants on obesity and T2D related outcomes in a Ghanaian population. Three hundred and two healthy Ghanaian adults (25–60 years old) from Oforikrom, Municipality in Kumasi, Ghana were randomly recruited and had genetic tests, dietary consumption analysis, and anthropometric and biochemical measurements of glucose, HbA1c, insulin, cholesterol, and triglycerides taken. A significant interaction was identified between vitamin D-GRS and fiber intake (g/day) on BMI (*p*_interaction_ = 0.020) where those who were consuming low fiber (≤16.19 g/d) and carrying more than two risk alleles for vitamin D deficiency (*p* = 0.01) had a significantly higher BMI. In addition, an interaction between vitamin D-GRS and fat intake (g/day) on HbA1c (total fat, *p*_interaction_ = 0.029) was found, where participants who had a lower total fat intake (≤36.5 g/d), despite carrying more than two risk alleles, had significantly lower HbA1c (*p* = 0.049). In summary, our study has identified novel gene–diet interactions of vitamin D-GRS with dietary fiber and fat intakes on metabolic traits in Ghanaian adults.

## 1. Introduction

There is an observed increase in the prevalence of obesity and Type 2 diabetes (T2D) in Sub-Saharan African (SSA) countries, and their related chronic diseases which are becoming a rising cause of morbidity and mortality [1,2,3]. Ghana is a West African country with a public health concern of an increase in obesity and overweightness, which is mainly attributed to rapid urbanization along with increased industrialization, use of motorized transport, increased income, a Westernized diet, and reduced physical activity [4,5,6]. The prevalence of the combined percentage of overweightness and obesity in Ghana was reported to be around 43% in a systemic review meta-analysis in 48,966 adults (mean age range: 23–56.2 years old) from ten regions in Ghana [7]. Likewise, in 2018, the prevalence of T2D in Ghanaian adults has been estimated to be in the range of 6.2–13.9% [8] with a substantial proportion of undiagnosed cases that typically are identified with the onset of diabetic complications [9,10]. Various factors predispose individuals to develop obesity and T2D including older age, diet, and inactivity. Additionally, genome-wide association studies (GWAS) in diverse populations have discovered more than 1100 loci to be associated with obesity traits and nearly 600 loci to be associated with T2D risk, suggesting the role of genetic factors in metabolic diseases [11,12].

Vitamin D deficiency status has been demonstrated to be influenced by several genetic factors, including genes involved with its synthesis and metabolism [13,14]. The 7-dehydrocholesterol reductase gene (*DHCR7*) encodes the enzyme that converts 7-dehydrocholesterol (7DHC) to cholesterol, [15,16,17], and the 25-hydroxylase gene (*CYP2R1*) encodes the enzyme that converts vitamin D to the circulating form 25(OH)D [16,17,18]. Vitamin D metabolism genes include the 24 hydroxylase gene (*CYP24A1*), which encodes the enzyme that catabolizes the biologically active form of vitamin D (1,25-dihydroxyvitamin D) to the inactive and water-soluble calcitroic acid, the excretory product of vitamin D metabolism, thus, controlling the amount of active vitamin D in the blood [16,17,19], the vitamin D binding protein (*DBP*)/group-specific component gene (*GC*), which binds to 25-hydroxyvitamin D and its plasma metabolites to transport them to target tissues [16,17,20], and the vitamin D receptor gene (*VDR*), which encodes the nuclear vitamin D receptor and heterodimerizes with the retinoid X receptor (*RXR*) to enable the biological activities of 1,25-dihydroxyvitamin D [21,22]. Finally, the calcium sensing receptor gene (*CASR*), which is important in regulating calcium homeostasis and synthesis of the parathyroid hormone (PTH), has been shown to stimulate the synthesis of 1,25-dihydroxyvitamin D [23,24]. Numerous studies have identified genetic variants associated with vitamin D status and those genetic markers have been validated as genetic instruments for low vitamin D concentrations [13,16,21].

Several epidemiological studies investigating the link between vitamin D status and metabolic traits have reported inconsistent findings where some studies found an association between vitamin D deficiency and metabolic outcomes [25,26,27] while other studies failed to find any connection [25,26,27,28]. Using genetic variants to examine the relationship between vitamin D status and metabolic disease outcomes has been shown to be an effective method to overcome confounding [29]. Therefore, the aims of this study were to use a nutrigenetic approach to determine whether a genetic risk score (GRS) of eight single nucleotide polymorphisms (SNPs) from six selected candidate genes related to vitamin D deficiency was associated with metabolic disease-related traits and whether these associations were modified by dietary intake in 302 randomly chosen healthy adults from Oforikrom, Ghana.

## 2. Methodology

### 2.1. Study Population

Study population was taken from the Genetics of Obesity and Nutrition in Ghana (GONG) study, which is a cross-sectional study in healthy Ghanaian adults aged 25–60 years. The study which took place in Oforikrom Municipality in Kumasi, Ashanti region, Ghana. The GONG study is a part of the Gene–Nutrient Interactions (GeNuIne). Collaboration, the main objective of which is to explore gene–nutrient interactions on metabolic disease outcomes in different ethnicities using population-based studies from various countries [30,31,32,33]. The Oforikrom Municipal Assembly is one of the 43 districts in the Ashanti region in Ghana. Oforikrom was a part of the Kumasi Metropolitan Assembly until 2018 when it was elevated to a municipal assembly district. In the Oforikrom Municipal Assembly there are seventeen recognized communities with an estimated total population of 360,254. Five communities (Oforikrom, Ayeduase, Ayigya, Kotei, and Bomso) were randomly selected from the list of communities in the Oforikrom Municipal Assembly. In each community, a central point was located (automobile station, marketplace, or a landmark). An assigned field investigator chose to enter the first house that is facing either East, West, North or South of that central point. After selecting the house, the investigator requested to randomly recruit one person from the household. If no one agreed to participate in the household, the investigator moved on to the next household. Subsequently, the fieldworker entered the next house, and the selection process was repeated. All participants freely agreed to participate using a written informed consent.

**Figure 1:**
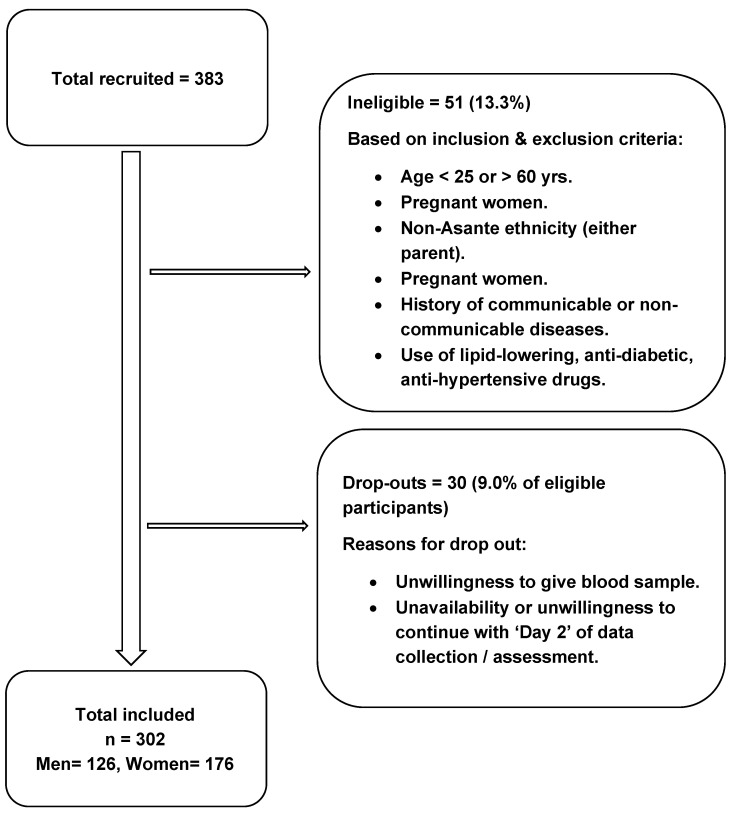
Flow chart showing the recruitment of the study participants.

A total of three hundred and two healthy free-living and adult volunteers were included in this study. Participants had no prior diagnosis of disease or physical complaints; all participants were screened and recruited for the study by trained investigators (Figure 1). A pre-questionnaire was developed and used where participants were asked if they had been diagnosed with cancer, diabetes, high cholesterol, high blood pressure, inflammatory, respiratory, gastrointestinal, thyroid, renal, liver or heart diseases, and about medication use and recent surgeries, to eliminate any unhealthy volunteers. The inclusion criteria were: (a) age from 25 to 60 years old; (b) healthy adults; and (c) both parents to be Asante ethnicity. The exclusion criteria were: (a) age less than 25 years or above 60 years; (b) pregnant women; (c) current diagnosis or having a history of communicable disease or any non-communicable diseases such as, cardiovascular diseases, T2D, and hypertension; and (d) use of medication for controlling diabetes, and hypertension, or lipid-lowering drugs.

Ethical approval was obtained from the Council for Scientific and Industrial Research (CSIR) Institutional Review Board (IRB) (Ref: RPN 003/CSIR-IRB/2018). Furthermore, this study was approved by the Metro Director of Health Services, Kumasi (KMHD/MPHs/13). An informed consent form was signed by each participant prior to their participation in the study.

### 2.2. Data Collection

Questionnaires were used to gather information about the participants’ demographic characteristics, dietary intakes, sleep, sunshine exposure patterns, and medical history, and demographic characteristics. Proper training was provided to the field investigators prior to the start of data collection. The instruments used for the survey were pre-tested to make sure that investigators had complete understanding of the questionnaires. The study was conducted from July to September 2018.

Anthropometric data [weight, height, hip circumference (HC), waist circumference (WC), visceral fat, and body fat percentage (BFP)] were measured with participants wearing light clothing. Height was measured to the nearest 0.1 cm, with a stadiometer (Seca 213 mobile stadiometer, Hamburg, Germany) with participants standing upright without shoes. Weight was measured to the nearest 0.1 kg, using an OMRON Body Composition Analyzer which also provided the values for the visceral fat, BFP, and body mass index (BMI). Non-extensible measuring tape was used to measure HC and WC. The HC was measured at the level of the greater trochanter to the nearest 0.1 cm whereas the WC was measured just above the naval to the nearest 0.1 cm. Waist-to-hip ratio (WHR) was calculated by dividing WC by HC.

### 2.3. Biochemical Measurements

Blood samples were collected via venipuncture after an 8–12 h fast from each participant by a trained phlebotomist. Fasting plasma glucose, glycated haemoglobin (HbA1c), fasting insulin, and lipid profile were analyzed. Fluoride tubes and ethylene–diaminetetra acetic acid (EDTA) tubes were used to collect blood samples which were stored in ice packs for temporary storage and transported to the Clinical Analyses Laboratory, KNUST for analysis. Blood glucose was analyzed using a semi-automated spectrophotometer (Biolabo Diagnostic Kenza Biochemistry Try, France). Fasting serum insulin was analyzed using an enzyme-linked immunosorbent assay (ELISA) kit. Ion-exchange chromatography method was used to determine total glycated hemoglobin. Total cholesterol, HDL-c, and serum triglycerides concentrations were determined by using a semi-automated spectrophotometer (Humalyzer Junior, Human GmBH Germany). The Friedewald formula was used to compute concentration of LDL-c in all the samples. All test kits were from Medsource Ozone Biomedicals Pvt. Ltd. Haryana, India

## 3. Assessment of Dietary Intake

A repeated three-day 24-h dietary recall (one weekend day and two weekdays) was used to extract the participants’ dietary intake data. The participants were asked to recall time of all meals taken and all the content of the consumed meals. Common household measures were used to help participants accurately estimate the actual amounts of foods and drinks consumed. An African specific dietary software (Nutrient Analysis Template; Food Science and Nutrition Department, University of Ghana, Accra, Ghana, 2010) was then used to analyse the nutritional composition of the participants’ diet.

### SNP Selection, GRS Construction and Genotyping

The following 8 SNPs were selected based on their known association with vitamin D concentrations: *VDR* SNPs rs2228570 and rs7975232 [24,34]; *DHCR7* SNP rs12785878 [24,35,36,37,38]; *CYP2R1* SNPs rs12794714 and rs10741657 [21,24,37,39,40]; *CYP24A1* SNP rs6013897 [24,36,40]; *DBP*/*GC* SNP rs2282679 [35,39]; *CASR* SNP rs1801725 [24,41]. Selected gene variants were tested using a goodness-of-fit Chi-squared test and all were in Hardy–Weinberg equilibrium (HWE) (*p* > 0.05) (Supplementary Table S1).

The collected blood samples for the DNA analyses were transported in dry ice to the United Kingdom (UK). Subsequently, genomic DNA extraction took place from a 5 mL whole blood sample from each participant, and genotyping was performed using the competitive allele-specific PCR-KASP assay at the LGC Genomics (http://www.lgcgroup.com/services/genotyping, accessed on 26 June 2022).

Vitamin D-related GRS was constructed by the addition of the sums of the risk allele across each of the eight SNPs. Given that there are no studies pertaining to vitamin D risk allele data in the West African population, the risk alleles for the present study were determined based on studies that were carried out in Caucasian populations [34,42,43,44,45,46,47,48,49,50,51,52]. Each single SNP was assigned a value of 0, 1, or 2 and this value designates the number of risk alleles. Subsequently, these values were calculated by the addition of the total risk alleles across each SNP and the score ranged from 0 to 6 risk alleles. Risk allele scores were then divided by the median, which was 2 risk alleles, and categorized into a “low genetic risk group” and a “high genetic risk group.” By use of the median of vitamin D-related GRS, low risk and high risk were categorized as individuals carrying less than 2 risk alleles (*n* = 68) and those carrying 2 or more risk alleles (*n* = 211), respectively. The risk alleles were not weighted, since no previously reported effect sizes were available for these SNPs for Ghanaians, and weighting of risk alleles has shown to have only limited effects [53]. Sample size calculations could not be performed because there were no previously reported effect sizes available for these SNPs for Ghanaians.

## 4. Statistical Analysis

Analyses of data were performed using Statistical Package for the Social Sciences (SPSS) software (version 27; SPSS Inc., Chicago, IL, USA). Descriptive characteristcs of study population were given as means and standard deviations (SD) for continuous variables, and between group comparisons were tested using an independent samples t-test. The Shapiro–Wilk test of normality was performed on continuous variables to test if the variables were in normal distribution. All variables were non-normally distributed; therefore, natural log transformation values were used in association and interaction analyses for all variables. General linear models (GLM) were used to analyze the association between vitamin D-related GRS and biochemical and clinical metabolic outcomes (BMI, WC, WHR, BFP, glucose, HbA1c, fasting insulin). The vitamin D-GRS and dietary intake interactions were also analyzed using GLM by incorporating the interaction terms in these models. Models were adjusted for age, gender, and BMI (when BMI was not an outcome), and total energy intake (only in the nutrient–GRS interaction analysis). The dietary factors investigated in our study included total dietary intake of carbohydrate, protein, fat, and fiber. If the interaction of the GRS with total fat intake was significant, further analyses were performed to assess the influence of saturated fatty acids (SFA), monounsaturated fatty acids (MUFA), and polyunsaturated fatty acids (PUFA). All vitamin D-GRS and dietary interactions reaching a nominal level of significance were further investigated using a binary and tertile stratification of dietary factors. The two-tailed value of *p* < 0.05 was considered statistically significant.

## 5. Results

### 5.1. Characteristics of Study Participants

GONG study participants were stratified by gender, and their baseline characteristics of clinical, anthropometric, biochemical, and dietary measurements were compared and summarized in Table 1. The mean BMI and total energy intake of all participants were 26.6 ± 5.0 kg/m^2^ and 1645 ± 688 kcal, respectively. Several significant differences in clinical, anthropometric, and dietary measurements were identified between men and women. Women had a significantly higher age, BMI, WC, and BFP than men (*p* < 0.003). Conversely, men had a significantly higher total energy intake and higher dietary intake of all measured nutrients: protein, carbohydrates, fat, SFA, MUFA, PUFA and fiber (*p* < 0.006). However, there were no significant differences in biochemical measurements (glucose, HbA1c and fasting insulin) between women and men.

### 5.2. Genetic Associations between Vitamin D-GRS and Metabolic Traits

No statistically significant associations were found between vitamin D-GRS and anthropometric and biochemical measurements (*p* > 0.12 for all comparisons) (Supplementary Table S2).

### 5.3. Interactions between Dietary Factors and Vitamin D-GRS on Metabolic Traits

**Figure 2:**
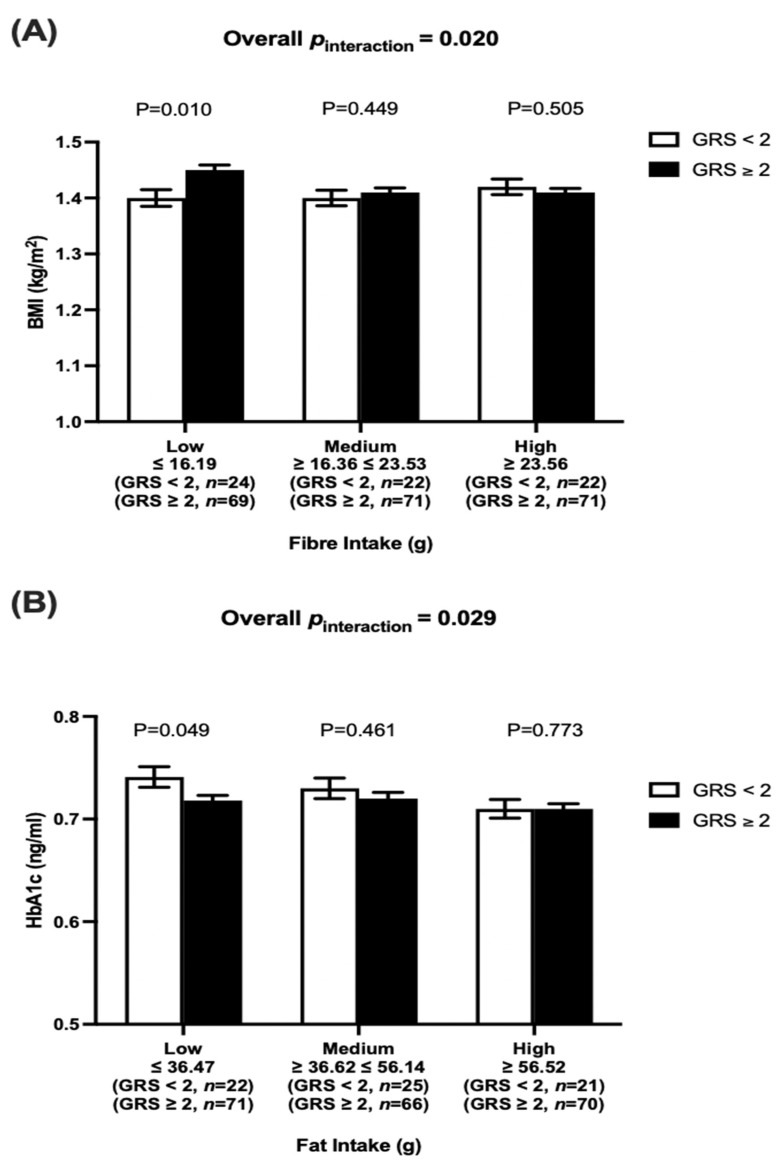
(**A**) Interaction between vitamin D-GRS and total dietary fiber intake (g) on log BMI (*p*_interaction_ = 0.020); Participants who consumed lower fiber (≤16.19 g/d) and carried ≥2 risk alleles (mean ± SE: 1.45 ± 0.009) had significantly higher BMI than participants with <2 vitamin D risk alleles (mean ± SE: 1.40 ± 0.015). (**B**) Interaction between vitamin D-GRS and total fat intake (g) on log HbA1c (*p*_interaction_ = 0.020); Participants who consumed lower fat intake (≤36.47 g/d) and carried ≥2 risk alleles (mean ± SE: 0.72 ± 0.005) had significantly lower HbA1c than participants with <2 risk alleles (mean ± SE: 0.74 ± 0.010).

There was a statistically significant interaction between vitamin D-GRS and fiber intake (g/day) on BMI (*p*_interaction_ = 0.020). Participants who consumed low fiber (≤16.2 g/d) and carried more than two risk alleles (mean ± SE: 1.45 ± 0.009, *p* = 0.010) had a significantly higher BMI than participants with less than two vitamin D risk alleles (mean ± SE: 1.40 ± 0.015, *p* = 0.010) (Figure 2A).

There was also a significant interaction between vitamin D-GRS and fat intake (g/day) on HbA1c (*p*_interaction_ = 0.029), where participants who had a lower consumption of dietary fat (≤36.5 g/d) and carried more than two risk alleles (mean ± SE: 0.72 ± 0.005, *p* = 0.049) had a significantly lower HbA1c than participants with less than two risk alleles (mean ± SE: 0.74 ± 0.010, *p* = 0.049) (Figure 2B). Total fat was stratified into SFA, MUFA, and PUFA and we found that SFA showed a significant interaction (*p*_interaction_ = 0.044) with vitamin D-GRS on HbA1c (Table 2). However, after dividing participants based on SFA intake (low SFA, medium SFA, and high SFA), no significant differences were detected between participants with high or low vitamin D deficiency genetic risk in any of the fat intake groups.

## 6. Discussion

To the best of our knowledge, our study is the first to use a nutrigenetic approach to examine the interaction between vitamin D genetic variants and dietary factors on metabolic outcomes in healthy West African adults from Ghana. This study demonstrated the association of vitamin D-GRS on BMI, a diagnostic marker of obesity [54,55], through the interaction with dietary fiber intake. Additionally, this study provided evidence for an interaction between vitamin D-GRS and fat intake on HbA1c levels, an indicator of glycemic control [56,57]. Both these findings are in agreement with general dietary recommendations addressing dietary fiber and total dietary fat consumption [58] to decrease the risk of obesity and T2D [59,60,61,62,63]. Our results carry public health implications where individuals with genetic risk of vitamin D deficiency might benefit from adhering to general dietary recommendations to prevent obesity and T2D.

According to the Institute of Medicine (IOM), the dietary guidelines for total daily fiber intake is 14 g/1000 kcal/day and, for total daily fat intake, the recommendation is between 20–35% of total energy intake [58]. In our cohort, the average total energy intake was 1645 kcal/d; hence the recommended average fiber intake should be 23 g/day, and the total recommended fat intake should be between 36.5–64.0 g/day. The findings from our study confirms the importance of adhering to the dietary fiber and fat intake guidelines and emphasizes their importance to individuals with a higher genetic predisposition to vitamin D deficiency to prevent obesity. Our study has demonstrated a significant impact of vitamin D-GRS on BMI which was detected under the influence of a low-fiber diet (≤16.2 g/day); hence increasing the dietary fiber consumption will benefit those individuals who are genetically susceptible. Likewise, reducing the consumption of dietary fat intake (<20 g/day) might be beneficial in improving the glycemic control in genetically susceptible individuals.

The results of our study emphasize the importance of a high fiber diet for individuals who have genetic risk of vitamin D deficiency. The role of dietary fiber in relation to the vitamin D-GRS and BMI is not clear; however, both fiber and vitamin D are involved in the health and regulation of the gut microbiome [64]. Dietary fiber is known to have helpful effects on weight management due to their bulking effect and fermentation by the gut microbiota [65,66]. Prebiotics specifically, when consumed, induce changes in intestinal microbiota diversity and increase the release of short-chain fatty acids (SCFAs), such as butyrate, which has the capacity to regulate anti-inflammation processes [67,68]. Obesity is known to be associated with chronic inflammatory markers, most notably the overexpression of pro-inflammatory cytokines produced in adipose tissues [69]. Furthermore, obesity is associated with altered gut microbiota composition and/or activity in humans [70]. As for vitamin D, studies have demonstrated that vitamin D and its receptor, VDR, can regulate and influence gut microbiota [71,72]. Vitamin D has immune-modulatory properties such as inhibiting inflammation and infections and hence might be important in modifying gut microbiota [64]. As both fiber and vitamin D have anti-inflammatory properties, and both are potential modifiers of the gut microbiome, therefore, our gene–fiber intake interaction on BMI could be possibly a result of the influence of microbiome modulation brought about by reduced dietary fiber intake in participants with an increased genetic susceptibility to vitamin D deficiency. Furthermore, some studies have examined the impact of dietary fiber and genetic markers on obesity outcomes. One study in Asian Indian adults (*n* = 1618) reported that fiber intake and fat mass obesity gene (*FTO*) SNPs had a significant influence on BMI and WC (*p*_interaction_ = 0.0008), where participants with high fiber intake (44 g/day) had a lower BMI (*p* = 0.07) and lower WC (*p* = 0.02) [73]. Another study reported a significant interaction between metabolic-related GRS and fiber intake on BFP, where participants with a higher fiber intake (>19 g/day) had lower BFP [74].

A recent longitudinal observantional study (*n* = 2500) reported that dietary fiber consumption in Ghanaians (24.9 ± 9.7 g/day) was higher than in Jamaicans, Seychellois, South Africans and Africans from the United States, which could be attributed to the high consumption of cassava in Ghana [75]. Nevertheless, only 43% of the study participants [75] met the recommended dietary guidelines for fiber intake (14 g/1000 kcal/day) established by the Institute of medicine (IOM) [58], and fiber intake continue to decline with rapid urbanization in Ghana [76]. It is established that higher total fiber intake is associated with lower inflammation, obesity, and BMI [58,77]. However, studies focusing on the relationship between fiber intake and obesity in the African population are limited. A recent cross-sectional study in 406 Ghanaian men (20–29 years old) demonstrated a significant inverse relationship between fiber intake and abdominal obesity [78]. Furthermore, a randomized trial in 107 African American women investigating fiber intake after 6- and 18-month dietary weight loss interventions found that the consumption of fiber at the 6-month follow-up was significantly and negatively associated with BMI, and a stronger negative association was found at the 18-month follow-up BMI [79]. It is important to emphasize the importance of increasing fiber intake for individuals at risk of vitamin D deficiency to meet the recommended dietary guidelines to help tackle the current rise in obesity in Ghana.

Nutritional transition from traditional diet to a Westernized diet plays a role in the increasing dietary fat consumption in SSA countries, and Ghana is no exception [76,80]. The effects of the changing diet from a low-fat rural diet to a high fat urban diet have already translated in to increased obesity and T2D in the Ghanaian population [3,81]. A recent nutrigenetics study in healthy Ghanaian adults (*n* = 302) [74] found a significant interaction between metabolic-related GRS and high total fat intake (>47 g/day) and SFA (>14 g/day) on increased WC, suggesting the existence of an interplay between fat intake and genetic susceptibility on obesity outcomes and supporting the general dietary recommendations to reduce total fat intake and SFA to prevent obesity. Our study showed that a lower fat intake was associated with a better glycemic control for people with an increased genetic risk of vitamin D deficiency. Links between total fat intake and glycemic control have been reported in previous studies on diabetic participants where one crossover clinical trial in diabetic adults (*n* = 7) from Israel reported that higher dietary fat intake increased glucose concentrations [82]. Additionally, a cross-sectional study in 150 adult participants from New Zealand found that increased SFA was associated with a 6% (95% CI 2–10%; *p* = 0.004) increase in HbA1c and concluded that reducing SFA maybe helpful in improving glycemic control [83]. Total fat intake seems to have an effect on HbA1c levels; this could be caused by reduced insulin sensitivity, or it could be because higher fat intake is causing an increased hepatic glucose production, which can cause an increase in the peak time and amount of the glucose response [84,85]. Evidence that dietary fat intake can affect glycemic control in vitamin-D-susceptible individuals has important implications for developing strategies to prevent T2D in this subgroup.

Our study has several strengths, of which being the first nutrigenetic study to examine the interaction between vitamin D-related genetic variants of and metabolic traits in healthy West Africans from Ghana is the main strength. In addition, we used the GRS approach which increased the statistical power of our analysis, and reduced the negative impact of multiple testing [21,86]. Furthermore, anthropometric and biochemical measurements were determined using validated techniques by skilled staff. Population stratification was also reduced as the participants were strictly Asante Ghanaians (both parents). Limitations include the small sample size; however, we were still able to identify significant gene–diet interactions, indicating that the study is sufficiently powered to detect significant interactions. We used a repeated 24-h dietary recall method to assess nutrient intake, which could be biased or have some recall errors. The study design is cross-sectional, which is inherently prone to residual confounding [87,88]. Finally, the study population were of a specific ethnic group in Ghana (Asante) which may not represent other ethnic groups in Ghana. Hence, more studies are required to confirm our findings to establish the clinical significance and possible applications as part of metabolic disease prevention.

## 7. Conclusions

In conclusion, the current study has identified novel gene–diet interactions in the West African Ghanaian population. Our study has shown that low fiber intake was associated with higher obesity and low fat intake was associated with greater glycemic control in vitamin D genetically susceptible individuals. Given that both obesity and T2D are on the rise in Ghana [7,9], our study highlights the importance of implementing strategies to follow IOM dietary guidelines to increase dietary fiber intake to 14 g/1000 kcal/day and to decrease total fat intake to 20% of total energy for genetically susceptible individuals. These gene–diet interaction findings need to be replicated in a larger cohort before any dietary recommendations can be implemented for genetically susceptible individuals.

## Figures and Tables

**Table 1:** Baseline characteristics of study participants.

	*n*	Total	*n*	Men	*n*	Women	*p* Value
Age (years)	279	38 ± 10	115	36 ± 9	164	40 ± 10	0.003
BMI (kg/m^2^)	279	26.6 ± 4.91	115	23.6 ± 3.02	164	28.7 ± 4.92	<0.001
WC (cm)	279	88.4 ± 12.22	115	81.8 ± 9.92	164	93 ± 11.59	<0.001
WHR	279	1.5 ± 7.24	115	0.9 ± 0.1	164	1.9 ± 9.43	0.15
BFP (%)	279	32.9 ± 13.55	115	20.5 ± 10.01	164	41.6 ± 7.58	<0.001
Glucose (mg/dl)	278	4.4 ± 0.91	115	4.3 ± 0.59	163	4.4 ± 1.09	0.33
HbA1c (%)	275	5.3 ± 0.58	111	5.3 ± 0.5	164	5.3 ± 0.62	0.94
Fasting Insulin (µIU/mL)	270	12.6 ± 14.38	109	13.1 ± 16.08	161	12.3 ± 13.15	0.62
Total Cholesterol (mg/dL)	276	212.7 ± 58	113	208.8 ± 41.76	163	216.6 ± 39.06	0.07
HDL-c (mg/dL)	276	69.6 ± 7.70	113	69.6 ± 7.35	163	65.7 ± 0.7.73	0.12
LDL-c (mg/dL)	276	127.6 ± 41.76	113	123.7 ± 42.54	163	131.5 ± 40.99	0.06
Serum Triglycerides (mg/dL)	276	87.3 ± 32.78	113	86.8 ± 29.23	163	87.7 ± 36.32	0.98
Total Energy Intake (kcal)	279	1645 ± 688	115	1901 ± 714	164	1465 ± 610	<0.001
Protein (g)	279	53 ± 23	115	63 ± 24	164	46 ± 19	<0.001
Carbohydrate (g)	279	240 ± 98	115	281 ± 104	164	211 ± 81	<0.001
Fat (g)	279	51 ± 27	115	57 ± 29	164	47 ± 24	0.001
Saturated fat (g)	279	16 ± 10	115	18 ± 11	164	15 ± 9	0.006
Monounsaturated fat (g)	279	18 ± 10	115	20 ± 11	164	16 ± 9	0.002
Polyunsaturated fat (g)	279	9 ± 5	115	10 ± 6	164	8 ± 5	0.002
Dietary Fibre (g)	279	22 ± 11	115	25 ± 12	164	19 ± 10	<0.001

Data are presented as means ± SD, *p* values for the differences in the means between the two groups were calculated by using the independent samples *t*-test, Abbreviations: BMI: body mass index, WC: waist circumference, WHR: waist–hip ratio; BFP: body fat percentage; HbA1c: glycated hemoglobin; HDL-c: high-density lipoprotein cholesterol; LDL-c: low-density lipoprotein cholesterol.

**Table 2:** Interaction between dietary factors and vitamin D-GRS on clinical and metabolic traits.

	Carbohydrates (g)	Protein (g)	Fat (g)	Fibre (g)	SFA (g)	PUFA (g)	MUFA (g)
BMI (kg/m^2^)	0.05	0.16	0.99	0.02			
WC (cm)	0.16	0.07	0.22	0.13			
WHR	0.72	0.76	0.85	0.87			
BFP (%)	1.00	0.27	0.22	0.12			
Glucose (mg/dL)	0.98	0.83	0.88	0.52			
HbA1c (ng/mL)	0.06	0.12	0.03	0.10	0.04	0.13	0.84
Fasting Insulin (µIU/mL)	0.35	0.68	0.43	0.13			

GLM was used to perform interaction analysis. All variables were log transformed. All associations were adjusted for age, gender, BMI (except BMI which was not adjusted for when the outcome was BMI) and total energy. The analysis was conducted on log-transformed variables. Abbreviations: GRS: genetic risk score, BMI: body mass index, WC: waist circumference, WHR: waist–hip ratio, BFP: body fat percentage, HbA1c: glycated hemoglobin.

## Data Availability

The datasets used and/or analysed during the current study are available from the corresponding author on reasonable request.

## Supplementary Materials

The following supporting information can be downloaded at: https://www.mdpi.com/article/10.3390/nu14132763/s1, Table S1: Genotypic and allelic frequencies of the single nucleotide polymorphisms that were used to create the genetic risk score. Reference [89] was cited in Table S1. Table S2: Genetic associations of vitamin D-GRS with clinical and biochemical measurements.

## Abbreviations

SSA	Sub-Saharan Africa
T2D	type 2 diabetes
WC	waist circumference
BMI	body mass index
HbA1c	glycated haemoglobin
*VDR*	vitamin D receptor
*DHCR7*	7-dehydrocholesterol reductase
*CYP2R1*	25-hydroxylase
*CYP24A1*	24-hydroxylase
*DBP*	vitamin D binding protein
*GC*	group-specific component
*CASR*	calcium sensing receptor
GRS	genetic risk score
SNP	single nucleotide polymorphism
HWE	Hardy Weinberg equilibrium
SD	standard deviation
IOM	Institute of Medicine
