# Supplementary material for: Interactions between Vitamin D Genetic Risk and Dietary Factors on Metabolic Disease-Related Outcomes in Ghanaian Adults
Source: Nutrients. 2022 Jul 4;14(13):2763. doi: 10.3390/nu14132763 (PMC9269445; doi:10.3390/nu14132763)
Supplement: Supplementary file 1 [file nutrients-14-02763-s001.zip › nutrients-1759150-supplementary.pdf]

Supplementary Table S1. Genotypic and allelic frequencies of the single nucleotide polymorphisms that were used to create the genetic risk score

	Single Nucleotide Polymorphisms	Nucleotide Change	Total	Common Homozygous	Heterozygote	Rare Homozygous	Hardy Weinberg Equilibrium *p* Value	Minor Allele Frequency	dbSNP* Frequency in Africans AFR
VDR	rs2228570	T/C	279	TT: 4	TC: 87	CC: 188	0.08	T= 0.17	T=0.31250000
	rs7975232	C/A	279	CC: 29	CA: 123	AA: 127	0.92	C= 0.32	C=0. 35929999
DHCR7	rs12785878	T/G	279	GG: 221	TG: 55	TT: 3	0.84	T= 0.11	T= 0.17019999
CYP2R1	rs12794714	A/G	279	GG: 230	GA: 46	AA: 3	0.68	A= 0.09	A= 0.10290000
	rs10741657	A/G	270	AA: 21	AG: 100	GG: 158	0.35	A= 0.25	A=0.21860000
CYP24A1	rs6013897	A/T	279	TT: 157	TA: 100	AA: 22	0.28	A= 0.26	A= 0.26019999
GC	rs2282679	C/A	279	AA: 263	CA: 16	CC: 0	0.62	C= 0.03	C= 0.04990000
CASR	rs1801725	T/G	279	GG: 27474	TG: 5	TT: 0	0.88	T= 0.01	T= 0.00980000

*dbSNP database: https://www.ncbi.nlm.nih.gov/snp/ [1]

References

1. National Centre for Biotechnology Information. https://www.ncbi.nlm.nih.gov/snp/.

Supplementary Table S2. Genetic associations of vitamin D-GRS with clinical and biochemical measurements

Clinical and Biochemical Parameters	GRS < 2		GRS ≥ 2		
	n	**Mean±SE**	***n***	**Mean±SE**	***p* Value**
BMI (kg/m2)	68	1.41± 0.008	211	1.42± 0.005	0.12
WC (cm)	68	1.94± 0.003	211	1.94± 0.002	0.88
WHR	68	-0.04± 0.021	211	-0.05± 0.012	0.66
BFP (%)	68	1.48± 0.014	211	1.47± 0.008	0.43
Glucose (mg/dl)	68	0.64± 0.008	210	0.63± 0.004	0.24
HbA1c (ng/ml)	68	0.73± 0.006	207	0.72± 0.003	0.20
Fasting Insulin (uIU/ml)	64	0.98± 0.034	206	0.98± 0.019	0.98

All variables were log transformed. All associations were adjusted for age, gender, total energy intake and BMI (except BMI which was not adjusted for when the outcome was BMI). Abbreviations: GRS: genetic risk score, BMI: body mass index, WC: waist circumference, WHR: waist-hip ratio, BFP: body fat percentage, HbA1c: glycated haemoglobin.
